# Can Exercise Training Improve the Quality of Life and Physical Function in Multiple Myeloma Patients?: Discussing the Progression of the Training Stimulus

**DOI:** 10.3390/sports14020061

**Published:** 2026-02-04

**Authors:** Polyxeni Spiliopoulou, Evangelos Eleutherakis-Papaiakovou, Magdalini Migkou, Nikolaos Kanellias, Ioannis Ntanasis-Stathopoulos, Panagiotis Malandrakis, Foteini Theodorakakou, Despina Fotiou, Pantelis Rousakis, Chrysanthi Panteli, Evangelos Terpos, Maria Gavriatopoulou, Ourania E. Tsitsilonis, Efstathios Kastritis, Meletios Athanasios Dimopoulos, Gerasimos Terzis

**Affiliations:** 1 Sports Performance Laboratory, School of Physical Education and Sport Science, National and Kapodistrian University of Athens, 17237 Athens, Greece; gterzis@phed.uoa.gr; 2 Department of Clinical Therapeutics, School of Medicine, Alexandra General Hospital, National and Kapodistrian University of Athens, 17237 Athens, Greece; 3 Flow Cytometry Unit, Department of Biology, National and Kapodistrian University of Athens, 17237 Athens, Greece; 4 Department of Medicine, Korea University, Seoul 02841, Republic of Korea

**Keywords:** multiple myeloma, quality of life, exercise training, handgrip strength, bone density, physical function

## Abstract

Background: Previous studies have reported no improvements in quality of life or physical function following exercise training in patients with multiple myeloma, without a clear explanation. The purpose of the present study was to examine the effects of an exercise-training intervention on these outcomes and to determine whether the observed results could be explained by the characteristics of the training program. Methods: Sixteen patients with multiple myeloma who had completed first-line induction therapy were assigned to two groups. One group (2 men, 6 women, 52.6 ± 10.3 years) commenced maintenance therapy only, while the other group (2 men, 6 women, 58.8 ± 7.1 years) initiated maintenance therapy combined with a supervised exercise training program conducted twice per week. Each exercise session included 30 min of cycling and seven resistance exercises targeting the major muscle groups. The intervention lasted 4.4 ± 1 months. QoL, the six-minute walking test, handgrip strength, maximal aerobic power, maximum isometric contraction across 14 positions, and bone density were assessed before and after the intervention. Results: The six-minute walking test distance significantly increased in the exercise group (9.36 ± 6.88%, *p* = 0.001), while no change was observed in the control group (3.34 ± 5.68%, *p* = 0.162); however, the difference between groups was not statistically significant (*p* = 0.076). QoL and maximal aerobic power remained unchanged in both groups, while maximal isometric strength increased significantly in both groups. Conclusions: The progression of the training stimulus appears to be inconsistent in this specific population, potentially limiting improvements in quality of life and physical function outcomes. Supervision appears to be necessary for exercise training in patients with multiple myeloma. Future research should investigate alternative exercise modalities in these patients.

## 1. Introduction

Multiple myeloma is a type of cancer in the plasma cells of the bone marrow [1]. The increased infiltration of malignant plasma cells into the bone marrow disrupts the body’s normal physiological functions. Initially, normal hematopoiesis is impaired, particularly the production of red blood cells, resulting in anemia [2,3]. Moreover, disease-related mechanisms stimulate osteoclast activity while inhibiting osteoblast function, leading to the formation of osteolytic lesions in patients’ bones [4]. Subsequently, impaired bone metabolism prevents the proper binding of calcium within bone tissue, causing elevated calcium levels in the bloodstream (hypercalcemia) [5]. Finally, the excessive production of paraprotein—a pathological protein secreted by myeloma cells—obstructs the renal tubules, leading to renal insufficiency [6]. These manifestations, collectively known by the acronym CRAB (hyperCalcemia, Renal failure, Anemia, Bone lesions), contribute to the deterioration of both quality of life and physical function in patients with multiple myeloma [7].

Structured exercise programs have been shown to improve quality of life and physical function in patients with cancer [8,9]. Systematic exercise interventions have also been applied to patients with multiple myeloma; however, it remains unclear whether such programs are beneficial for this population [10]. Specifically, a recent meta-analysis reported that exercise has not been found to produce significant changes in quality of life among patients with multiple myeloma [11]. Similarly, the distance achieved in the six-minute walking test increased in both the control and exercise groups, and handgrip strength did not change in either group following a 10-week exercise intervention, which included 20 min of aerobic exercise at 12–13 RPE and 3 sets of 12–15 repetitions for 9 resistance training exercises, in newly diagnosed multiple myeloma patients [12]. The same study also found that knee extension strength decreased in the exercise group but remained unchanged in the control group, while bone mineral density remained unchanged after the exercise training [12]. In accordance with these findings, handgrip strength did not alter, and VO_2_max was not improved after 6 months of 30 min aerobic training at 50–75% of HRmax combined with resistance training exercises in multiple myeloma patients following maintenance therapy [13].

Although exercise has occasionally led to benefits, such as improvements in lower limb strength [13], the overall evidence does not yet provide clear support for the efficacy of exercise in patients with multiple myeloma. Before drawing definitive conclusions, greater attention should be given to the specific exercise programs implemented in this population and to a more detailed examination of what occurs during training. Continuous supervision of patients during exercise sessions and systematic documentation of how training parameters are adjusted to individual needs are essential for accurately assessing the impact of exercise interventions in this context.

Previous studies have found no improvements in quality of life or physical function following exercise training in patients with multiple myeloma, which may be related to the progression of the training stimulus. Thus, the aim of this study is to examine the effects of a fully supervised structured exercise program on quality of life, physical function, and bone mineral density in patients with multiple myeloma, and to interpret the findings by analyzing what occurs during the training period. This discussion will be of particular importance for the development of exercise prescription guidelines for patients with multiple myeloma.

## 2. Materials and Methods

### 2.1. Experimental Design

A total of sixteen patients diagnosed with multiple myeloma participated in the study. The exercise group included eight patients who engaged in a structured exercise program with supervision in addition to receiving standard medical treatment. The control group also comprised eight patients who received medical therapy only. The study commenced 100 days after autologous stem cell transplantation. The end of the intervention was determined in conjunction with scheduled clinical assessments (e.g., blood tests and bone marrow aspiration), in accordance with the physicians’ instructions. Consequently, the duration of the intervention varied among participants and lasted 4.4 ± 1 months (Table 1). The quality of life, physical function and bone density were analyzed before and after the intervention period.

### 2.2. Participants

Multiple myeloma patients were recruited from the Alexandra General Hospital in Athens, Greece, between November 2021 and April 2023. Patients who met the inclusion criteria were asked to participate in the present study. In total, 16 participants accepted participation and performed both the initial and final measurements (Table 1). The inclusion criteria required that all participants (i) had completed their first-line induction therapy, (ii) undergone ASCT, (iii) were at the initiation stage of maintenance treatment and (iv) had an Eastern Cooperative Oncology Group (ECOG) Performance Status of 0–2. All participants provided written informed consent after being fully briefed on the experimental procedures and voluntarily agreed to participate. The study protocol was reviewed and approved by the Bioethics Committee of the Institutional Review Board (project number 1202/10-06-2020) and was conducted in accordance with the ethical standards of the World Medical Association’s Declaration of Helsinki (1964, revised in 2013).

### 2.3. Training Intervention

Exercise training was performed at the gym of the School of Physical Education and Sport Science in Athens. Only one participant was at the gym during training. Sessions were not scheduled at a fixed time of day but were arranged according to participants’ availability. All training sessions were supervised by the same qualified exercise professional. Although no physician was present during the sessions, all participants underwent a cardiological evaluation prior to enrollment and received medical clearance for exercise training. In addition, blood pressure was assessed at the beginning of each session, and participants were instructed to postpone training if abnormal values were detected (>150/90 mmHg and <100/70 mmHg); however, this situation did not occur during the intervention period. Participants completed at least 70% of the training sessions.

Training was conducted twice per week, with a minimum interval of two days between sessions, over a period of 4.4 ± 1 months (range: 3–6 months). Each session lasted approximately 90 min and began with a 5 min warm-up on a stationary bicycle at an intensity of 25 watts. This was followed by 30 min of interval cycling and a full-body resistance training program, as described in detail below.

Peak aerobic power was assessed using an incremental aerobic test on a stationary recumbent cycle (see maximal aerobic power), conducted one week prior to the start of the training program. The aerobic training consisted of three sets of 10 min of continuous cycling at 80% of the peak heart rate on the recumbent bike, with a 2 min rest between sets, corresponding to approximately 12–13 on the Borg RPE 6–20 scale [14]. During training, the training intensity was monitored according to RPE scale only. If the RPE fell below 11, the workload was increased by 25 watts to raise the RPE to the target range of 12–13.

One repetition maximum was not performed in multiple myeloma patients [15] and RPE scale (6–20) was used to determine the intensity of resistance training. For each exercise, the load corresponding to an RPE of 12–13 was initially established [14]. Resistance training was conducted using a stationary multifunction resistance machine (Toorx MSX 70, Garlando S.p.A., Pozzolo Formigaro, Italy) and consisted of three sets of ten repetitions, with 1–2 min of rest between sets and 2–3 min between exercises. The program included seven exercises performed in the following sequence: triceps extension, elbow curl, lat pulldown, knee extension, bench press, hamstring curl, and lateral raise. When the perceived exertion dropped below 11, the load was increased by 1–3 kg to restore the RPE to 12–13.

### 2.4. Quality of Life

Health-related quality of life was assessed using the QLQ-C30 version 3.0 questionnaire, which is recommended by the European Organization for Research and Treatment of Cancer for use in cancer patients. Scores range from 0 to 100, and three main domains were evaluated: (a) global health status, (b) functional scales (physical, role, emotional, cognitive, and social functioning), and (c) symptoms (fatigue; nausea and vomiting; pain; dyspnea; insomnia; appetite loss; constipation; diarrhea; and financial difficulties). For domains (a) and (b), higher scores indicate better overall health and functional status, whereas for domain (c), higher scores reflect greater symptom burden or worse health-related problems.

### 2.5. Bone Density

Dual-energy X-ray absorptiometry (DXA) was used to evaluate bone mineral density (Lunar Prodigy, General Electric Medical Systems, Madison, WI, USA). Following calibration, participants were positioned supine within the DXA scanning frame. Scans were performed for the whole body, lumbar spine, and femur. Data analysis was conducted using Lunar Prodigy software (Prodigy Encore, version 18, General Electric, Madison, WI, USA). The following parameters were measured: total mass, total fat mass, and bone mineral density for the whole body, lumbar spine (L2–L4), and femur. The intraclass correlation coefficient (ICC) for measurements of body composition and bone density was 0.99.

### 2.6. Six-Minute Walking Test

The six-minute walking test was conducted indoors along a long, spacious corridor. Two cones were positioned 20 m apart, directly opposite each other. Participants began at one cone, walked to the other, made a counterclockwise turn, and continued back and forth for a total duration of six min. Time feedback was provided by the researcher at one-min intervals. Participants were instructed to walk as far as possible at a comfortable, continuous pace and were encouraged to notify the researcher if they experienced any discomfort or wished to stop. All participants, however, completed the six-minute walk without incident. The total distance covered was recorded and used for subsequent analysis. The ICC for this measurement was 0.95.

### 2.7. Handgrip Strength Test

Handgrip strength of both the right and left hands was assessed using a calibrated hand-held dynamometer (Camry, Model EH101, Zhongshan, China). Participants stood with their elbow fully extended during testing. Two trials were conducted for each hand, with a 30 s rest interval between attempts. The highest value from each hand was recorded, and the combined sum of right- and left-hand strength was used for analysis. The ICC for this measurement in our laboratory is 0.96.

### 2.8. Isometric Strength

Isometric strength was assessed using an isometric dynamometer (MicroFET 2 Wireless Manual Muscle Tester, Hoggan Scientific LLC, Salt Lake City, UT, USA; sampling rate: 100 samples per second) for both the right and left extremities. Participants performed isometric contractions at seven positions on each side: (1) shoulder adduction, (2) shoulder abduction, (3) hip flexion, (4) hip abduction, (5) hip adduction, (6) knee extension, and (7) knee flexion. Two maximal attempts were allowed at each position, with a one-min rest between trials, and the highest value was recorded. The sum of all muscle contractions was used as a measure of isometric strength in subsequent analyses.

### 2.9. Maximum Aerobic Power

The aerobic test was conducted on a recumbent stationary cycle ergometer (Ergociser, Model EC-3500, Cateye, Osaka, Japan) following the modified ACSM cycle ergometer protocol [16]. Participants began cycling at a workload of 25 watts and a cadence of 50 rpm, with the resistance increasing by 25 watts every three mins. The test was terminated once participants were unable to maintain the required pedaling frequency. Heart rate was continuously monitored throughout the test (Polar A300, Polar Electro Oy, FI90440, Kempele, Finland), and the heart rate corresponding to the final completed stage (peak aerobic power) was used to prescribe and regulate aerobic training intensity.

### 2.10. Statistical Analysis

Data are presented as mean and standard deviation (mean ± SD). According to the Kolmogorov–Smirnov test, all variables were normally distributed except for quality of life parameters. Statistical significance between the pre- and post-measurements was tested using a 2 × 2 ANOVA for repeated measures. Also, an independent samples *t*-test was performed to compare the percentage changes between the control and exercise groups. For the quality of life parameters, the non-parametric Wilcoxon test and the Mann–Whitney test were used accordingly. Significance was accepted at *p* ≤ 0.05 using a two-tailed test design. SPSS software version 21 was used for statistical analysis.

## 3. Results

### 3.1. Patients Characteristics and Feasibility

The characteristics of participants in both groups, along with the medical treatments received during maintenance therapy, are presented in Table 1. The intervention durations did not differ between groups (*p* = 0.117). All participants completed the training program twice a week without any adverse events, pain, or other problems. In contrast, they took part with great eagerness and thoroughly enjoyed it.

### 3.2. Quality of Life

The quality of life parameters were not changed after the intervention in both groups. Specifically, Global Health remained unchanged in the exercise group (pre: 65.28 ± 17.81%, post: 69.45 ± 11.38%, *p* = 0.593) and the control group (pre: 73.81 ± 8.91%, post: 72.92 ± 8.62%, *p* = 0.564), with no difference between groups (*p* = 0.434). Physical function was preserved in the exercise group (pre: 72.22 ± 12.23%, post: 83.33 ± 10.11%, *p* = 0.057) and the control group (pre: 77.14 ± 22.06%, post: 83.33 ± 12.34%, *p* = 0.216) with no difference between groups (*p* = 0.220). Role functioning remained unaltered in both the exercise (pre: 63.89 ± 30.58%, post: 83.33 ± 21.08%, *p* = 0.066) and the control group (pre: 76.16 ± 30.24%, post:83.33 ± 19.92%, *p* = 0.416) without difference between groups (*p* = 0.467). Emotional functioning was not changed after the exercise (pre: 86.11 ± 15.51%, post: 79.17 ± 25.69%, *p* = 0.357) or the control intervention (pre: 89.29 ± 18.46%, post: 86.46 ± 18.87%, *p* = 0.715) with no difference between groups (*p* = 0.606). Cognitive function was not altered during the exercise (pre: 88.89 ± 20.18%, post: 86.11 ± 12.55%, *p* = 0.713) and the control condition (pre: 86.11 ± 12.55%, post: 87.50 ± 23.15%, *p* = 0.705) without between-groups difference (*p* = 0.601). Social functioning was left unchanged in the exercise group (pre: 61.11 ± 17.21%, post: 69.45 ± 22.15%, *p* = 0.588) but it was significantly reduced in the control group (pre: 64.29 ± 17.82%, post: 93.75 ± 8.63%, *p* = 0.017) with no difference between groups (*p* = 0.128). Fatigue was not altered in the exercise (pre:29.63 ± 35.60%, post: 31.48 ± 19.14%, *p* = 0.916) and the control group (pre: 26.98 ± 19.09%, post: 26.39 ± 10.18%, *p* = 0.666) with no difference between groups (*p* = 0.942). The Nausea and Vomiting score was zero for all patients in both time points, except for two patients in the control group. For one participant, the score was 0% before the intervention and 50% after the intervention. For the other case, the score was 33.33% both before and after the intervention. No changes were made for the Pain scores in the exercise (pre: 16.67 ± 18.26%, post: 16.76 ± 18.62%, *p* = 0.999) and the control group (pre: 14.29 ± 14.99%, post: 14.58 ± 16.52%, *p* = 0.414) with no difference between groups (*p* = 0.745). Dyspnea was not changed in both the exercise (pre: 27.78 ± 25.09%, post: 38.89 ± 32.77%, *p* = 0.593) and the control group (pre: 33.33 ± 19.25%, post: 33.33 ± 17.82%, *p* = 0.999) with no difference between groups (*p* = 0.499). No difference was found for the exercise (pre: 16.67 ± 27.89%, post: 22.22 ± 40.37%, *p* = 0.785) and the control group (pre: 14.28 ± 17.82%, post: 20.83 ± 35.36%, *p* = 0.655) for Insomnia scores, with no difference between groups (*p* = 0.744). Appetite loss score was zero for all patients before and after the intervention, except for two participants. One participant in the exercise group (pre: 0%, post: 33.33%) and one participant in the control group (pre: 33.33%, post: 0%). Constipation was preserved as it was for the exercise (pre: 5.56 ± 13.61%, post: 11.11 ± 17.21%, *p* = 0.317) and the control group (pre: 14.29 ± 26.23%, post: 16.67 ± 35.63%, *p* = 0.318) with no changes between groups (*p* = 0.909). Diarrhea stayed unaltered for both the exercise (pre: 5.56 ± 13.61%, post: 22.22 ± 17.21%, *p* = 0.180) and the control group (pre: 0.00 ± 0.00, post: 4.17 ± 11.78, *p* = 0.317), with no difference between groups (*p* = 0.199). Finally, Financial Difficulties were not changed after the intervention for both the exercise (pre: 22.22 ± 27.22%, post: 11.11 ± 17.21%, *p* = 0.317) and the control (pre: 14.28 ± 17.82%, post: 4.17 ± 11.78%, *p* = 0.083) group, with no changes between groups (*p* = 0.482).

### 3.3. Physical Function

The six-minute walking test distance (m) was significantly increased in the exercise group (9.36 ± 6.88%, *p* = 0.001) while it was not changed in the control group (3.34 ± 5.68%, *p* = 0.162), with no significant difference between groups (*p* = 0.076). Isometric handgrip strength of the right hand did not alter in the exercise group (pre: 35.66 ± 10.79 kg, post: 35.91 ± 11.57 kg, *p* = 0.839) or the control (pre: 36.94 ± 9.68 kg, post: 38.69 ± 8.03 kg, *p* = 0.716), with no significant difference between groups (*p* = 0.198). Isometric handgrip strength of the left hand remained unchanged for the exercise group (pre: 34.10 ± 10.73 kg, post: 33.94 ± 12.02 kg, *p* = 0.896) and the control group (pre: 35.84 ± 5.87 kg, post: 37.11 ± 6.84 kg, *p* = 0.313) with no difference between groups (*p* = 0.389). The sum of all isometric contractions increased significantly in both the exercise group (pre: 198.50 ± 44.22 kg, post: 226.23 ± 54.27 kg, *p* = 0.018) and the control group (pre: 198.99 ± 64.93 kg, post: 221.51 ± 51.30 kg, *p* = 0.047) with no significant difference between groups (*p* = 0.857). The maximum aerobic power remained similar after the intervention for the exercise group (pre: 112.50 ± 29.88 watt, post: 121.88 ± 47.13 watt, *p* = 0.276) and the control group (pre: 96.88 ± 28.15 watt, post: 100.00 ± 35.36 watt, *p* = 0.432), with no significant difference between groups (*p* = 0.734).

### 3.4. Bone Density

Total mass, fat mass and bone density of the total body, spine and femur for both groups, before and after the intervention, are presented in Table 2.

## 4. Discussion

The aim of this study was to examine the effects of supervised combined aerobic and resistance training on physical function and quality of life in patients with multiple myeloma who had completed first-line therapy and autologous transplantation and were beginning maintenance treatment. All participants successfully completed the training program without any difficulties or injuries. The feasibility of exercise training in patients with multiple myeloma has been previously discussed [17,18,19].

The main finding of the study is that quality of life did not improve in either group. The time point at which a patient may experience the most pronounced symptoms of multiple myeloma and the lowest quality of life is at diagnosis, due to the manifestation of serious complications such as fractures, anemia, or renal insufficiency [20]. The present study, however, initiates the intervention after the patients have completed their initial therapy, at which point the disease has either fully or substantially regressed. Consequently, patients’ quality of life may have already returned to relatively higher levels, making further improvements difficult to detect over the following months, even with the addition of an exercise training program. In addition, the present training protocol did not seem to increase the strength or the aerobic power of the patients (see below), which also explains the maintenance of life quality. Possibly, a different training protocol could be more suitable for multiple myeloma patients. Previous studies have not provided evidence of improvement for the quality of life after systematic exercise in patients with multiple myeloma [11].

Maximal aerobic capacity did not change in either group. More specifically, within the exercise group, maximal aerobic capacity increased in three patients, remained stable in four, and decreased in one. Among the three patients who improved, the progressive increase in workload followed a consistent pattern, approximately 25 watts every 2–3 weeks. In the four patients whose capacity remained unchanged, workload progression was considerably slower (approximately 25 watts every 5–6 weeks), and on occasions when they reported feeling more fatigued, they reverted to the previous workload for one or two sessions. The patient whose aerobic capacity declined had anemia as a primary disease-related symptom. From the third month of training onwards (total training duration 5 months), this individual experienced worsening anemia, which made aerobic training more difficult and ultimately necessitated a reduction in workload. Nevertheless, progressive increases in aerobic workload are essential for improving performance in both healthy individuals [21,22] and clinical populations. In patients with coronary disease, aerobic exercise intensity was increased every 4 weeks, resulting in improvements in heart rate reserve, indicating enhanced cardiovascular fitness [23]. Similarly, in patients with neuromuscular disorders, aerobic workload was progressively increased over a 16-week training period when Borg RPE values were below 13, leading to improvements in maximal aerobic power [24]. The findings of the present study highlight that, in patients with multiple myeloma, the principle of progressive overload in aerobic training is not easily achieved. A different aerobic training modality, like high-intensity interval training, has not been investigated in this population. Possibly, the intervals could be more suitable for the patients, and the intensity could be more easily increased. Nevertheless, all patients continued aerobic exercise despite the minor adjustments in cycling workload, without needing to interrupt the program, missing sessions, or experiencing any adverse effects. A previous study likewise demonstrated that aerobic capacity did not improve after three or six months of combined aerobic and resistance training [13].

In addition, although resistance training loads were progressively increased to maintain a constant intensity corresponding to 13 on the Borg scale, the progression was relatively slow. ACSM guidelines for the progressive load of resistance training recommend load increases of approximately 2.5–10% per week [25]. Consistent with these recommendations, strength-training programs in healthy individuals typically apply weekly load increments of 2–2.5%, resulting in improvements in one-repetition maximum performance [21,26]. Similarly, in clinical populations, such as patients with neuromuscular disorders or pancreatic cancer, resistance-training loads have been increased by approximately 5% when participants were able to complete additional repetitions at a given load, leading to enhanced performance outcomes [24,27]. In contrast, in the present study, resistance loads were often maintained for longer periods, ranging from 2 to as many as 3–5 weeks. For example, one participant performed leg extensions at 20 kg for four consecutive weeks before the load was increased to 22 kg for three weeks. Another participant trained the triceps extension at 17 kg for five weeks, followed by an increase to 18 kg for three weeks. This suggests that muscular adaptations to resistance training in this patient population may occur more slowly compared with healthy individuals or other disease populations. It possibly implies that a different resistance training protocol, such as a muscle endurance training intervention, could be more suitable for these patients. A previous study similarly reported that insufficient progression of resistance-training load may explain the persistence of fatigue and the lack of improvement in quality of life in cachectic patients with head and neck cancer following 13 resistance-training sessions [28].

Supervision appears to be crucial for exercise training in patients with multiple myeloma, as the principle of progressive overload cannot always be consistently applied. Further research is needed to explore a wider range of exercise modalities and determine which are most suitable for this population, as well as which ones can effectively enhance quality of life, muscular strength, and aerobic capacity. It should also be documented that previous studies from our laboratory demonstrated that the current training protocol improved the immune microenvironment of the bone marrow in these patients [29], while a better bone marrow microenvironment is connected with better quality of life [30]. Both biological and performance-related parameters should be systematically examined across different exercise modalities to better understand their potential benefits.

The sum of isometric contractions increased similarly in both groups. The increase in isometric strength observed in the control group may be explained as a regaining of neural adaptations that had been reduced during the long period of inactivity following the autologous transplantation. However, the exercise intervention did not induce any additional improvement. It is possible that further gains in the exercise group might have been detected if maximal strength had been assessed through dynamic testing (1RM) rather than isometric measures, as this would more closely reflect the mode of exercise execution during training. Nevertheless, 1RM testing is not considered appropriate for evaluating maximal strength in patients with multiple myeloma [15].

Similarly, the handgrip strength did not change after the exercise intervention. Previous studies likewise have not demonstrated improvements in functional capacity, as indicated by strength-related parameters, following training interventions [13,18,31]. One study that initiated training at the time of diagnosis found that knee extension strength decreased in the exercise group following the intervention, while handgrip strength declined in both the exercise and control groups [12]. Similarly, six months of strength training or walking did not result in changes in handgrip strength [32].

Furthermore, improvements in the six-minute walking test were observed exclusively in the exercise group. The six-minute walking test is widely recognized as a valid and reliable measure of physical function in patients [33]. Importantly, an increase of more than 30 m is considered clinically significant [33]. In this study, the exercise group improved from a mean distance of 509 m to 550 m, thereby underscoring the clinical significance of the training intervention. This outcome was further supported by participants’ self-reports from the first month of training onwards, including statements such as “I can now climb stairs without holding onto the handrail” and “I feel my body is in better condition.” The observed improvement did not appear to be attributable to aerobic adaptations or increases in muscular strength. It may therefore be explained by other factors not assessed in the present study, such as neural adaptations or psychological parameters. A previous investigation examining the six-minute walking test after a 10-week training program reported similar improvements in both the exercise and control groups [12]. However, it should be emphasized that in that study the intervention began at diagnosis, including a combination of supervised and unsupervised sessions, and was of considerably shorter duration.

Resistance training has been shown to increase bone density in both healthy individuals [34] and patients with osteoporosis [35]. Patients with multiple myeloma often develop bone lesions and consequently reduced bone density [4], although no change in bone density was observed in the present study. This finding may be explained by the lack of progressive increases in training load during the intervention, as mentioned above. It should also be mentioned that the intervention period lasted only 3–6 months, which may have been insufficient to detect measurable differences in this parameter. Mechanical loading has been reported to decrease cortical porosity, increase cortical thickness, and enhance trabecular bone mass in the tibiae of mice with multiple myeloma [36]. Nevertheless, bone density did not increase after a 10-week exercise intervention in newly diagnosed multiple myeloma patients either [12].

The patients who participated in this study were in similar disease stages, but the total sample size was small, which is a limitation of the present experimental approach. In addition, participants received various medical treatments, and the intervention duration was different among patients. Although there is currently no clear evidence that different treatment regimens differentially affect quality of life or exercise performance, a more homogeneous sample receiving the same medical treatment and exercising for the same duration could strengthen the interpretation of the results. The long-term effects of exercise training cannot be determined from the present study. Longer interventions are required to evaluate the effects of exercise after several years, as well as the potential impact of maintaining an active daily lifestyle on these parameters. Moreover, the timing of intervention within the treatment trajectory—whether at diagnosis, before or after first-line therapy, or at the beginning or later stages of maintenance therapy—may lead to different outcomes.

## 5. Conclusions

In conclusion, the four-month training intervention implemented in the present study led to an increase in six-minute walking distance, possibly as a result of neural adaptations or psychological factors. However, it did not produce improvements in isometric strength, bone density, maximal aerobic power, or quality of life. These findings may be partly explained by the slow progression of resistance loads and the inconsistent progression of aerobic workloads during the training period. Future research should explore alternative exercise modalities, such as muscle-endurance resistance training and high-intensity interval aerobic training, in patients with multiple myeloma.

## Figures and Tables

**Table 1:** Participants’ characteristics.

	Exercise Group	Control Group
Sample size	N = 8	N = 8
Men	N = 6	N = 6
Women	N = 2	N = 2
Intervention duration (months)	4.9 ± 1	3.9 ± 1
Age (years)	58.8 ± 7.1	52.6 ± 10.3
Total mass (kg)	80.1 ± 18.8	88.9 ± 22.2
Height (m)	1.73 ± 0.1	1.75 ± 0.1
BMI (kg/m^2^)	26.6 ± 4.7	28.9 ± 6.8
Maintenance treatment		
Proteasome inhibitors		N = 3
Immunomodulatory drugs		N = 4
Proteasome inhibitors + Immunomodulatory drugs		N = 3
Anti-CD38 antibodies + Immunomodulatory drugs		N = 1
Anti-CD38 antibodies + Proteasome inhibitors		N = 2
Anti-CD38 antibodies + Immunomodulatory drugs + Proteasome inhibitors		N = 3

BMI: Body Mass Index; Anti-CD38 antibodies: anti-Cluster of Differentiation 38 monoclonal antibodies.

**Table 2:** Bone density before (PRE) and after (POST) about 4 months of exercise or control intervention in multiple myeloma patients following first-line treatment.

	Exercise Group				Control Group				
	PRE	POST	%DIFF	*p*	PRE	POST	%DIFF	*p*	*p*’
Total mass (kg)	80.13 ± 18.79	82.71 ± 20.60	3.06 ± 3.54	0.057	88.85 ± 22.18	90.04 ± 23.32	1.20 ± 3.47	0.371	0.308
Fat mass (kg)	26.99 ± 9.87	27.51 ± 12.17	0.89 ± 16.53	0.830	31.84 ± 10.32	29.79 ± 14.20	−7.61 ± 36.03	0.407	0.554
BMD total body (g/cm^2^)	1.17 ± 0.19	1.19 ± 0.17	1.92 ± 4.23	0.265	1.23 ± 0.13	1.20 ± 0.14	−2.50 ± 0.13	0.100	0.052
BMD spine (g/cm^2^)	1.13 ± 0.18	1.16 ± 0.21	1.77 ± 3.81	0.195	1.14 ± 0.24	1.12 ± 0.25	−1.23 ± 4.58	0.476	0.192
BMD femur (g/cm^2^)	1.00 ± 0.13	0.98 ± 0.13	−2.52 ± 4.10	0.695	0.94 ± 0.13	0.93 ± 0.16	2.04 ± 7.76	0.116	0.169

*p*: significance values for pre-post differences, *p*’: significance values for between-groups differences, %DIFF: percentage change in pre-post differences, BMD: bone mineral density.

## Data Availability

The data that support the findings of this study are not openly available due to reasons of sensitivity and are available from the corresponding author upon reasonable request.
